# Impact of response to electrical cardioversion before catheter ablation for persistent atrial fibrillation: a propensity score-matched analysis

**DOI:** 10.1093/ehjopen/oeaf084

**Published:** 2025-06-28

**Authors:** Márton Boga, Zoltán Salló, Gábor Orbán, Ferenc Komlósi, Anna Padisák, Patrik Tóth, Péter Perge, Vivien Klaudia Nagy, Edit Tanai, István Osztheimer, Béla Merkely, László Gellér, Nándor Szegedi

**Affiliations:** Heart and Vascular Center, Semmelweis University, Városmajor Street 68, Budapest 1122, Hungary; Heart and Vascular Center, Semmelweis University, Városmajor Street 68, Budapest 1122, Hungary; Heart and Vascular Center, Semmelweis University, Városmajor Street 68, Budapest 1122, Hungary; Heart and Vascular Center, Semmelweis University, Városmajor Street 68, Budapest 1122, Hungary; Heart and Vascular Center, Semmelweis University, Városmajor Street 68, Budapest 1122, Hungary; Heart and Vascular Center, Semmelweis University, Városmajor Street 68, Budapest 1122, Hungary; Heart and Vascular Center, Semmelweis University, Városmajor Street 68, Budapest 1122, Hungary; Heart and Vascular Center, Semmelweis University, Városmajor Street 68, Budapest 1122, Hungary; Heart and Vascular Center, Semmelweis University, Városmajor Street 68, Budapest 1122, Hungary; Heart and Vascular Center, Semmelweis University, Városmajor Street 68, Budapest 1122, Hungary; Heart and Vascular Center, Semmelweis University, Városmajor Street 68, Budapest 1122, Hungary; Heart and Vascular Center, Semmelweis University, Városmajor Street 68, Budapest 1122, Hungary; Heart and Vascular Center, Semmelweis University, Városmajor Street 68, Budapest 1122, Hungary

**Keywords:** Persistent atrial fibrillation, Catheter ablation, Electrical cardioversion, Pulmonary vein isolation, Recurrence

## Abstract

**Aims:**

We hypothesize that sinus rhythm (SR) maintenance in persistent atrial fibrillation (AF) patients taking anti-arrhythmic drugs (AADs) after pre-procedural electrical cardioversion (ECV) could predict outcomes after catheter ablation procedures.

**Methods and results:**

219 persistent AF patients on AADs underwent ECV 1–6 months before ablation. Patients were categorized into two groups according to their response to ECV: patients in whom SR was restored and maintained until the ablation procedure (ECV-SR group), and patients with AF recurrence before the procedure (ECV-AF group). Then, 1:1 propensity score matching was used to create study groups (94–94 patients). The efficacy outcomes of the present study were freedom from atrial tachyarrhythmia on/off AADs following a single ablation procedure and recurrence of persistent AF. The median follow-up duration was 42 (20–73) months. Freedom from atrial tachyarrhythmia at 36 months was lower in the ECV-AF group compared to ECV-SR patients (31.4% vs. 51.2%, respectively; crude HR = 2.58, 95% CI = 1.58–3.70, *P* < 0.001). The most frequent pattern of atrial arrhythmia recurrence was persistent AF in the ECV-AF group and paroxysmal AF in the ECV-SR group. Freedom from persistent AF at 36 months was 54% and 84.3%, respectively (crude HR = 3.72, 95% CI = 1.94–7.14, *P* < 0.001). Differences in the risk of the efficacy outcomes were similar after multi-variable adjustment and in all analysed subgroups, including pulmonary vein isolation (PVI)-only procedures.

**Conclusion:**

Our findings indicate that the positive response to pre-procedural ECV may be a valuable marker for identifying persistent AF patients in whom a PVI-only strategy is sufficient.

## Graphical Abstract

**Graphical Abstract:**
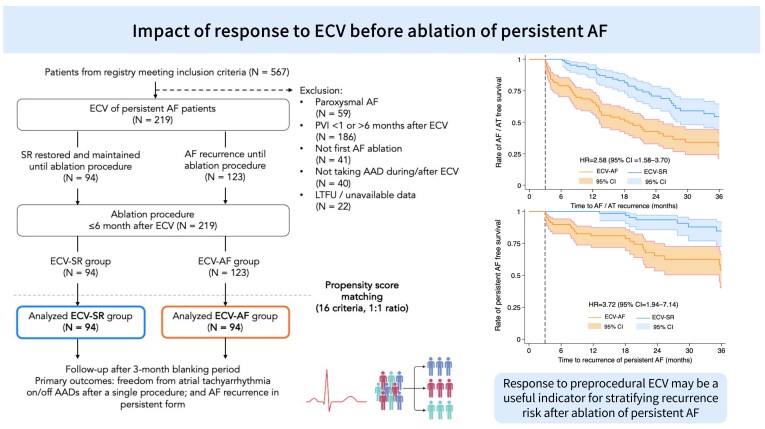

## Introduction

Atrial fibrillation (AF) is a progressive disease with a natural course characterized by increasing arrhythmia burden and progression from a paroxysmal to persistent form.^1^ This progression is associated with elevated risks for adverse outcomes such as ischaemic stroke, hospitalization for heart failure, or death.^2–4^ Concurrently, the likelihood of maintaining sinus rhythm (SR) diminishes, creating a vicious circle of worsening disease and therapeutic efficacy. Therefore, while pulmonary vein isolation (PVI) is proven effective for the treatment of persistent AF (PeAF),^5^ outcomes fall significantly short of those achieved in paroxysmal AF (PAF).^6^ Efforts to study substrate modification or extrapulmonary trigger ablation techniques in addition to PVI in PeAF patients have yielded mixed results.^7–12^ The latest guidelines classify the usefulness of such ablation strategies as an area of uncertainty,^6^ while according to survey results reported in the same document, one-third of operators routinely perform these techniques in PeAF ablation.^13^ However, it has to be recognized that AF patterns offer minimal insight into underlying mechanisms, such as the PV or extrapulmonary origin of triggers, and structural or electrical substrate remodelling. Consequently, their utility for patient selection is limited, potentially explaining the conflicting results observed in clinical trials with additional ablation techniques in all PeAF patients. There is limited guidance on identifying which patients should undergo PeAF ablation as first-line therapy or who would benefit from additional ablation beyond PVI.^5,6^ According to the recent EHRA survey, 40% of physicians consider the effectiveness of prior electrical cardioversion (ECV) when selecting candidates for ablation^13^; however, the small number of observational studies on this topic produced conflicting results about its utility in predicting the efficacy of procedures.^14–16^ We hypothesize that SR maintenance in patients taking anti-arrhythmic drugs (AADs) after pre-procedural ECV reflects the state of atrial electrical remodelling and therefore could be one of the strongest predictors of rhythm outcomes after ablation procedures. Hence, the goal of this study was to evaluate whether the response to ECV in PeAF patients could predict outcomes after catheter ablation.

## Methods

### Patient population

**Figure 1:**
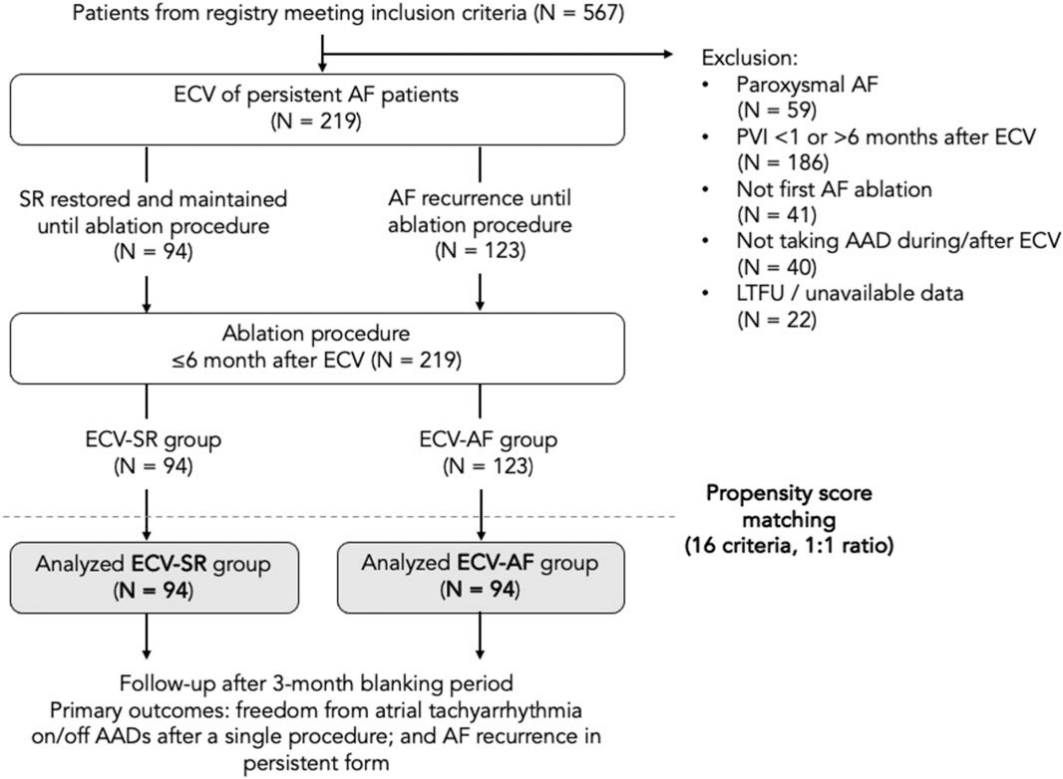
Study flowchart. AAD, anti-arrhythmic drug; AF, atrial fibrillation; ECV, electrical cardioversion; LTFU, loss to follow-up; PVI, pulmonary vein isolation; SR, sinus rhythm.

This retrospective propensity score matched (PSM) cohort study is based on data from an institutional registry containing prospectively collected information on consecutive procedures and follow-up of patients undergoing catheter ablation for AF at the Heart and Vascular Center of Semmelweis University, Budapest, Hungary. Inclusion criteria were catheter ablation 1–6 months after previous ECV between 2015 and 2022, and persistent AF at the time of ECV, defined as an AF episode lasting >7 days. Exclusion criteria were paroxysmal AF, repeat ablation, no AAD-effect during ECV/no AAD continuation until procedure, and follow-up < 6 months or unavailable data. Details of patient selection and creation of study groups is presented in the study flowchart (*Figure 1*). After applying inclusion and exclusion criteria, patients were categorized into two groups according to response to ECV: (i) patients in whom SR was restored and maintained until the ablation procedure (ECV-SR group, *n* = 94), and (ii) patients who have had AF recurrence and presented in AF at the procedure (ECV-AF, *n* = 123). Then, analysed study groups (both *n* = 94) were created by 1:1 PSM to account for potential confounding by baseline characteristics. All patients provided written informed consent for the storage and use of their data for research purposes. Ethical approval was obtained from the Semmelweis University Regional Research Ethics Committee (SE RKEB 25/2025).

### Outcomes

The primary outcomes of the present study were (i) freedom from AF and/or atrial tachycardia (AT) on/off AADs following a single ablation procedure, and (ii) recurrence of (or progression to) PeAF during follow-up, after a 3-month blanking period. Secondary outcomes included procedural complications; repeated catheter ablation; pulmonary vein (PV) reconnections at repeat procedures; and progression to permanent AF defined as AV-node ablation with pacemaker implantation, or PeAF accompanied by discontinuation of AADs and no further attempts at ECV or catheter ablation.

### Electrical cardioversion

All patients underwent transesophageal echocardiography (TEE) prior to ECV to rule out left atrial thrombus. Synchronized direct current shock was delivered under propofol anaesthesia, with an initial energy of 200 J. If SR was not restored, or AF restarted immediately, shocks with 360 J were applied. The procedure was discontinued after a maximum of three unsuccessful attempts with 360 J. Upon awakening, patients were assessed for neurological signs, and they were monitored for 6 h during which the potential recurrences were documented.

### Catheter ablation procedures

All patients took either amiodarone, propafenone, or sotalol before the procedures. Either TEE or contrast-enhanced left atrial computed tomography was used to exclude thrombi. Catheter ablation was performed under conscious sedation. After accessing the femoral vein, double transseptal puncture was applied under fluoroscopy guidance. An electroanatomical mapping system (either CARTO, Biosense Webster Inc., Diamond Bar, CA, USA; or ENSITE, Abbott Laboratories, Abbott Park, Illinois, USA) and a multi-polar mapping catheter were used to create an anatomical left atrial map. Wide, antral radiofrequency PVI was carried out with a point-by-point technique using a steerable sheath and a contact-force sensing ablation catheter. Once the initial circle was completed, the mapping catheter was used to check for gaps in the ablation line. Additional applications were delivered at these sites until complete PVI was achieved, confirmed by entrance and exit blocks in all PVs. Adjunctive ablation targeting empirical non-PV triggers (superior vena cava, vein of Marshall) and substrate modification (posterior wall isolation, other linear lesions) was performed rarely in selected cases. The decision to perform adjunctive ablation was made at the discretion of the operating physician, based on patient-related factors and intra-procedural findings, such as failure to terminate AF by PVI, or emergence of macroreentrant atrial tachycardias. Systematic voltage mapping or trigger induction protocols were not performed during procedures. Patients in AF at the end of the procedure were cardioverted to SR.

### Follow-up period

A 3-month blanking period was applied after the catheter ablation procedure, during which rhythm outcomes were not assessed. Atrial tachyarrhythmia recurrence (AF or AT/flutter of at least 30 s) was monitored in all patients by standard of care follow-up, with 12-lead electrocardiograms and 24 h Holter monitoring at 3-month visits in the first year and annually thereafter, or sooner in case of arrhythmia symptoms. The decision to discontinue AADs was left to the discretion of the treating physician. In the case of recurrence of atrial arrhythmias, AADs were reinitiated, and as a first step, patients were scheduled for ECV 4 weeks after the documentation of recurrence. If AF persisted until the scheduled visit, the cardioversion was performed and the recurrence was categorized as PeAF. The option for repeated ablation was evaluated by shared decision-making with the patient. During repeat procedures, PV reconnections were assessed, and PVs were reisolated. If AF persisted despite continued attempts at rhythm control, treating physicians could opt to discontinue AADs and pursue rate control including atrioventricular node ablation with pacemaker implantation. In such cases, the study endpoint of permanent AF was reached. The minimum duration of follow-up required to be included in this study was 6 month.

### Data collection

Patient characteristics (demographic information, disease history, medications, and TEE measurements), procedural and follow-up data were prospectively evaluated and collected in the institutional registry. These data were augmented by retrospective collection of missing information about patient characteristics and rhythm outcomes from electronic health records, and by contacting patients. Events of arrhythmia recurrence (both pre- and post-ablation) were registered in the case of atrial arrhythmia recorded by a 12-lead electrocardiogram, Holter monitoring, or cardiac implantable electronic devices. In the case of recorded recurrence, the type of atrial arrhythmia was categorized as paroxysmal AF, AT/AFL, or persistent AF (duration > 7 days). The progression of AF from paroxysmal to persistent (in the case of initial PAF recurrence) and from persistent to permanent form was also registered during data collection. The correct entry of study endpoints was monitored by a separate researcher blinded to study group allocation.

### Statistical methods

To create comparison groups, propensity scores were estimated with logistic regression based on the following 16 covariates: age, sex, CHA_2_DS_2_-VA score, left ventricular ejection fraction (LVEF), left atrial diameter (LAD), long-standing PeAF, hypertension, diabetes, chronic heart failure (CHF), valvular heart disease (VHD), coronary artery disease (CAD), stroke/transient ischaemic attack (TIA), additional ablation beyond PVI, time from ECV to ablation, year of ablation, and follow-up duration. Then patients were matched 1:1 using nearest neighbour PSM without replacements. The characteristics of the unmatched groups were compared with χ^2^-test or Fisher’s exact test in the case of binary variables, and the Mann–Whitney ranksum test in the case of non-parametric continuous or ordinal data. Characteristics of PSM groups were compared in a pairwise manner with McNemar’s test for binary data and the Wilcoxon signed-rank test for non-parametric continuous or ordinal data. Kaplan–Meier curves were generated to visualize cumulative incidence outcomes with 95% confidence intervals (CI). The log-rank test was used to compare freedom from AF between the matched groups. Multi-variable Cox proportional hazard regression was performed to eliminate remaining confounding effects and to assess the effect of other covariates in the outcome. Clinically relevant predictors (*P* < 0.25) or variables differing across matched groups were included in initial models. Then, a stepwise backward elimination method was used to exclude variables that did not influence the fit of the model. Schoenfeld’s residual test was performed to assess the proportional hazard assumption. Multiple imputation with 20 imputed datasets was used for missing values of LVEF (22% missing) and LAD (23% missing). Estimates were combined across datasets for the calculation of propensity scores and hazard ratios. Variables with missing values exceeding 25% were excluded from regression models. Subgroup analysis was performed to assess effect-modifications on the primary outcomes. *P*-values are reported for the primary outcomes and for comparison of baseline characteristics with the alpha level for significance set at 0.05. Statistical analyses were conducted using Stata (StataCorp LLC, College Station, TX, USA, release 18) and GraphPad Prism 10 (GraphPad Softwares Inc., San Diego, CA, USA) software.

## Results

### Characteristics of study groups

Characteristics of the unmatched population are presented in Supplementary material online, *Table S1*. After PSM, baseline characteristics were similar across study groups (*Table 1*), with a significant difference being the higher frequency of additional ablation beyond PVI in the ECV-AF group (19.1% vs. 6.4%, *P* = 0.023). In ECV-AF patients, SR could not be restored or AF immediately returned after ECV in 16 patients, and 7 had AF recurrence during the observation period after restoring SR. AF was documented in 38 patients between ECV and catheter ablation, while in 33 it was registered at the start of the procedure.

**Table 1:** Baseline characteristics of matched groups

	ECV-SR	ECV-AF	*P*-value
	*n* = 94	*n* = 94	
Age, years	63.5 (55–69)	62.5 (54–68)	0.827
Female sex, *n* (%)	23 (24.5)	28 (29.8)	0.442
BMI, kg/m^2^	29.3 (27.3–32.1)	29.4 (26.1–33)	0.901
Time since AF diagnosis, years	1.5 (0.6–5)	1 (0.5–3)	0.343
AF pattern			0.455
Persistent, *n* (%)	88 (93.6)	84 (83.4)	
Long-standing persistent, *n* (%)	6 (6.4)	10 (10.6)	
Hypertension, *n* (%)	56 (59.6)	65 (69.1)	0.243
CHF, *n* (%)	12 (12.8)	10 (10.6)	0.832
CAD, *n* (%)	9 (9.6)	14 (14.9)	0.405
VHD, *n* (%)	6 (6.4)	6 (6.4)	1
Stroke/TIA, *n* (%)	7 (7.4)	3 (3.2)	0.219
Diabetes, *n* (%)	14 (14.9)	20 (21.3)	0.362
PAD, *n* (%)	2 (2.1)	1 (1.1)	1
Hypertyreosis, *n* (%)	0 (0)	0 (0)	1
Left atrial diameter, mm	49 (45.5–52)	49 (43.5–52)	0.531
Right atrial diameter, mm	45.5 (41.5–49)	46 (39–51.5)	0.687
E wave velocity, cm/s	80 (63–­98)	93 (77–100)	**0**.**005**
LAA flow velocity, cm/s	39 (26–52)	30.5 (26–40)	0.093
LVEF, %	55 (53–60)	55 (53–60)	0.811
LAVI, mL/m^2^	46.1 (34.7–59.0)	46.2 (36.3–58.3)	0.981
CHA_2_DS_2_-VA score, *n* (%)			0.568
0	22 (23)	14 (15)	
1	26 (28)	32 (34)	
2	24 (26)	24 (26)	
3	15 (16)	14 (15)	
4	7 (7)	5 (5)	
5	0 (0)	4 (4)	
6	0 (0)	1 (1)	
Type of AAD before ablation			
Amiodarone, *n* (%)	78 (83)	83 (88)	0.460
Propafenone, *n* (%)	8 (8)	7 (7)	
Sotalol, *n* (%)	8 (8)	4 (4)	
ECV-to-PVI time, days	99.5 (71–130)	102 (69–134)	0.873
Date of ablation procedure, year	2018 (2016–2021)	2017 (2015–2020)	0.087
Procedure time, min	80 (70–110)	90 (70–110)	0.575
Left atrial dwell time, min	56.5 (43–75.5)	57 (42–70)	0.397
Additional ablation, *n* (%)	6 (6.4)	18 (19.1)	**0**.**023**
Posterior wall box lesion	3 (3.2)	9 (9.6)	
Mitral isthmus line	2 (2.1)	7 (7.4)	
CTI line	3 (3.2)	4 (4.3)	
CFAE ablation	0 (0)	0 (0)	
Follow-up duration, months	42 (22–71)	41 (17–75)	0.507

AAD, anti-arrhythmic drug; AF, atrial fibrillation; BMI, body-mass index; CAD, coronary artery disease; CFAE, complex fractionated atrial electrogram; CHF, chronic heart failure; CTI, cavotricuspid isthmus; ECV, electrical cardioversion; LAA, left atrial appendage; LVEF, left ventricular ejection fraction; PAD, peripheral arterial disease; PVI, pulmonary vein isolation; TIA, transient ischaemic attack; VHD, valvular heart disease. Continuous variables are presented as median (inter-quartile range) and compared with the Wilcoxon signed-rank test. Binary variables are presented as count (percentage) and compared with McNemar’s test. Bold *P*-values indicate significance at alpha < 0.05.

### Outcomes

**Figure 2:**
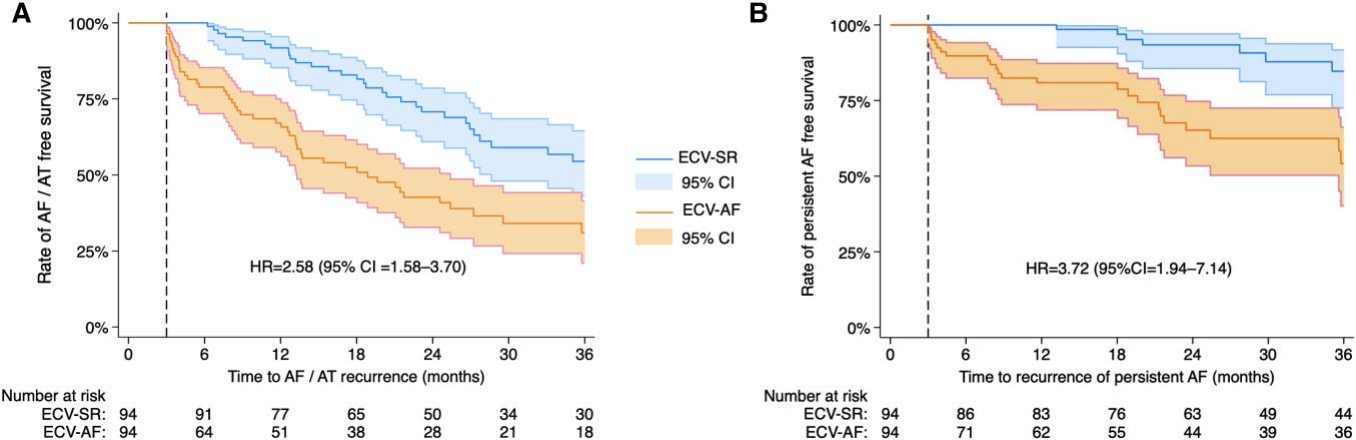
Kaplan–Meier curves with 95% CI for the rate of AF/AT free survival (*A*) and the rate of persistent AF-free survival (*B*) after a single procedure in the ECV-SR and ECV-AF groups. Dashed lines indicate the end of the 3-month blanking period. AF, atrial fibrillation; AT, atrial tachycardia; CI, confidence interval; HR, hazard ratio.

The median duration of follow-up was 42 (20–73) month in the overall cohort. The rate of freedom from AF/AT on/off AADs at 12 months was 91.7% in the ECV-SR group and 67% in the ECV-AF group; while at 36 months the rate was 51.2% and 31.4%, respectively (crude HR = 2.58, 95% CI = 1.58–3.70, *P* < 0.001; *Figure 2A*). The most frequent type of atrial arrhythmia recurrence was paroxysmal AF in the ECV-SR group and persistent AF in the ECV-AF group (*Table 2*). After multi-variable adjustment, ECV-AF (HR = 2.68, 95% CI = 1.71–4.20) was the only significant predictor of AF/AT recurrence following ablation (*Table 3*).

**Table 2:** Type of atrial arrhythmia recurrence in the ECV-SR and ECV-AF groups, presented as event numbers and column percentages

	ECV-SR	ECV-AF	RR (95% CI)
	*n* (%)	*n* (%)	
Recurrence of AF in persistent form	12 (40)	27 (57.5)	2.25 (1.24–4.17)
Recurrence of AF in paroxysmal form	16 (53.3)	15 (31.9)	0.94 (0.50–1.77)
Recurrence of AT/AFL	2 (6.7)	5 (10.6)	2.5 (0.58–11.0)

AF, atrial fibrillation; AFL, atrial flutter; AT, atrial tachycardia; CI, confidence interval, RR, risk ratio.

**Table 3:** Multi-variable cox-regression models

	Endpoint of AF/AT recurrence		Endpoint of AF recurrence in persistent form	
	HR (95% CI)	*P*-value	HR (95% CI)	*P*-value
VHD	1.86 (0.82–4.21)	0.139	1.98 (0.58–6.75)	0.275
Diabetes	1.67 (0.98–2.83)	0.058	0.61 (0.27–1.40)	0.242
Long-standing persistent AF	1.49 (0.67–3.34)	0.329	1.42 (0.40–5.02)	0.591
LVEF (%)	0.99 (0.96–1.22)	0.459	0.94 (0.90–0.98)	0.008
Additional ablation	0.63 (0.33–1.22)	0.171	0.40 (0.16–1.01)	0.052
ECV-AF	2.68 (1.71–4.20)	<0.001	6.15 (2.90–13.01)	<0.001

AF, atrial fibrillation; AT, atrial tachycardia; CI, confidence interval; HR, hazard ratio; LVEF, left ventricular ejection fraction; VHD, valvular heart disease.

The incidence rate for the recurrence of—or progression to—persistent AF was 7.5/100 patient-years (95% CI = 4.5–12.5) in the ECV-SR group and 22.8/100 patient-years (95% CI = 16.0–32.4) in the ECV-AF group. Freedom from persistent AF at 12 months was 100% and 80.6%; while at 36 months it was 84.3% and 54%, respectively (crude HR = 3.72, 95% CI = 1.94–7.14, *P* < 0.001; *Figure 2B*). In the multi-variable model (*Table 2*), significant predictors were AF recurrence after ECV (HR = 6.15, 95% CI = 2.90–13.01) and LVEF (measured in %, HR = 0.94, 95% CI = 0.90–0.98). Furthermore, additional ablation was at the threshold of significance associated with PeAF free survival (HR = 0.40, 95% CI = 0.16–1.01). Proportional hazard assumptions were met for all Cox-regression models (Schoenfeld test *P* > 0.9).

**Figure 3:**
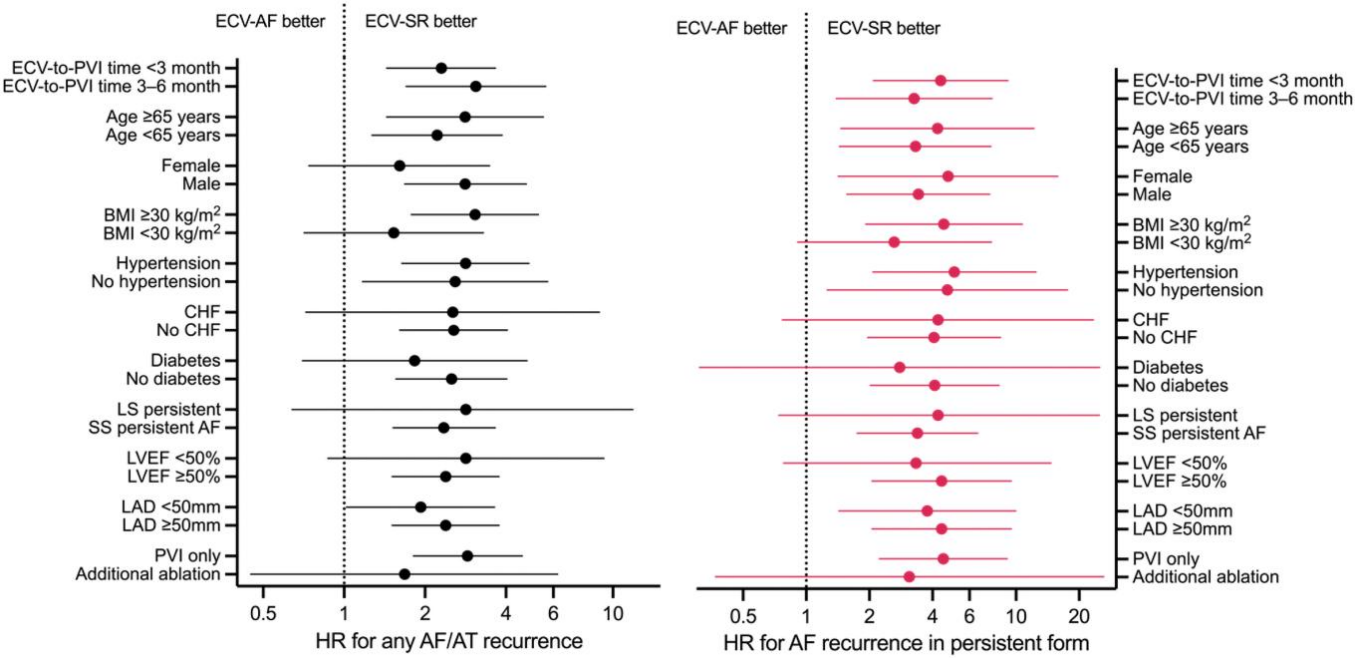
Hazard ratios for the effect of ECV response on the primary outcomes according to subgroups of patient characteristics and clinical parameters. AF, atrial fibrillation; AT, atrial tachycardia; BMI, body-mass index; CHF, chronic heart failure; HR, hazard ratio; LAD, left atrial diameter; LS, long-standing; LVEF, left ventricular ejection fraction; ECV, electrical cardioversion; PVI, pulmonary vein isolation; SS, short standing (duration < 1 year).

**Figure 4:**
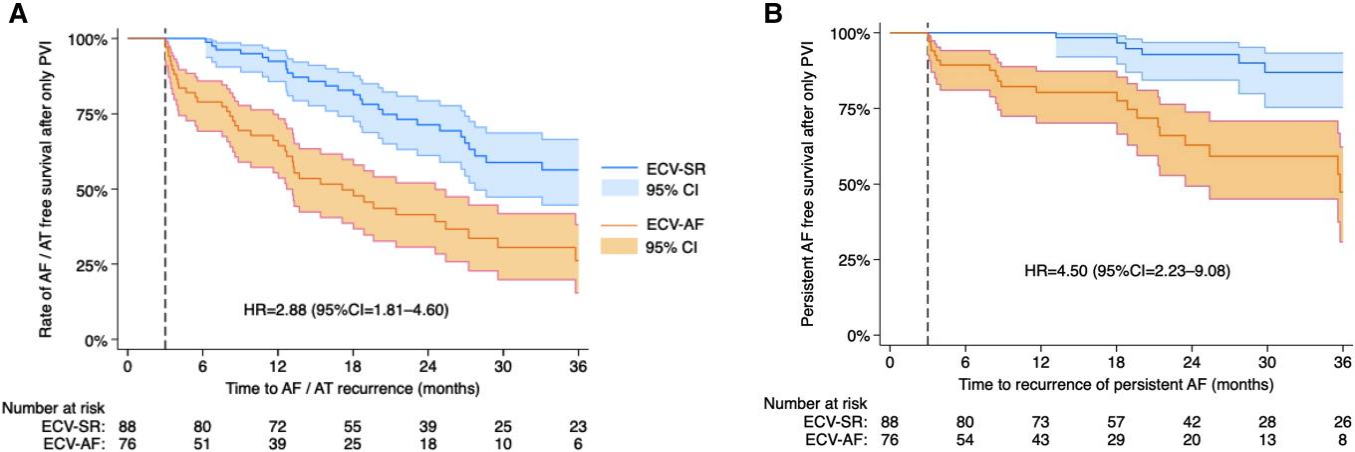
Kaplan–Meier curves with 95% CI for the rate of AF/AT free survival (*A*) and the rate of persistent AF-free survival (*B*) after a single procedure in the PVI-only group. Dashed lines indicate the end of the 3-month blanking period. AF, atrial fibrillation; AT, atrial tachycardia; CI, confidence interval; HR, hazard ratio.

Differences in the risk of the primary outcomes were similar in analysis of subgroups according to ECV-to-PVI time, age, sex, BMI, hypertension, CHF, diabetes, AF pattern, ablation strategy, LVEF, and LAD categories (*Figure 3*). Kaplan–Meier curves for the PVI-only subgroup are presented in *Figure 4*. The curves show a similar extent of separation as the main analysis, with HR = 2.88 (95% CI = 1.81–4.60) for AF/AT recurrence, and HR = 4.50 (95% CI = 2.23–9.08) for recurrence of persistent AF.

No major complication occurred as a result of ablation procedures. There were three cases of groin hematoma in the ECV-SR group not needing surgical repair. Repeated ablation was performed in 19 and 25 patients in the two groups (20% vs. 27%, RR = 1.32; 95% CI = 0.79–2.22), respectively. During repeat procedures, 52/75 (69.3%) PVs were found to be reconnected in ECV-SR patients and 56/93 (60.2%) in ECV-AF patients (RR = 0.77, 95% CI = 0.50–1.164). After multiple procedures, AF-free survival was similarly lower in the ECV-AF group, although the difference is somewhat less pronounced (HR = 1.83, 95% CI = 1.16–2.90, supplementary material online, *F*igure  *S1*). The rate of progression to permanent AF was 4.3% in the ECV-SR group and 10.6% in the ECV-AF group over the total follow-up (RR = 2.5, 95% CI =0.86–7.34).

## Discussion

The main observation of this study is that patients with PeAF on AADs, who maintain SR after ECV prior to ablation, have favourable rhythm outcomes after catheter ablation. Conversely, subjects with AF recurrence until the procedure have a significantly worse prognosis. The clinical relevance of this finding lies in the use of post-ECV SR maintenance as a simple and practical guide for identifying persistent AF patients in whom a PVI-only strategy may not be sufficient.

Existing literature on this topic includes a small number of retrospective observational studies. Kamada *et al*. categorized PeAF patients into two groups: those in whom SR was successfully restored by ECV (161 patients) and those in whom it was not (34 patients).^15^ They reported higher freedom from AF off AADs in the SR group. A recent study by Limite *et al*., the most comparable to ours, included 58 patients maintaining SR until the ablation procedure (at least 4 weeks from ECV), and 89 who reverted to AF after ECV.^16^ They report no difference in AF-free survival between groups. However, among patients presenting in SR at the procedure, PVI alone resulted in better freedom from AF than PVI + additional ablation. These patients also exhibited less frequent and less extensive low-voltage areas. Compared to these investigations, the novelty and strength of the present study are the following: (i) greater number of patients, (ii) propensity score-matched analysis, (iii) more rigorously defined patient selection criteria by exclusion of patients off AADs and ECV outside of 1–6-month pre-ablation time-window, and (iv) evaluation of endpoints also reflecting progression of AF.

Some other studies investigated similar research questions. One study observed that pre-ablation ECV shock energy and the number of shocks required to restore SR were higher in PeAF patients who experienced recurrences after ablation.^17^ Another investigation compared PeAF patients who maintained SR for at least 1 month after ECV with patients in whom ECV was not performed, and reported similar 12-month freedom from atrial tachyarrhythmia in the two groups, but concluded that the extent of ablation required to terminate AF during procedures is lower in the SR group.^14^ In an analysis of a long-standing PeAF population divided based on whether SR restoration with AADs and ECV was accomplished or not, lower rates of recurrence was observed in the SR group. Additionally, observational studies have highlighted the prognostic value of pre-procedural pharmacologic cardioversion with a similar effect as ECV.^18^ It can be concluded that the atrial rhythm before and during ablation procedures is an important predictor of procedural efficacy,^19^ and AF management strategies including AADs and ECV before the procedure can increase the accuracy and standardize this risk stratification approach.

The anticipated benefit of catheter ablation is a major factor influencing patient selection decisions.^20–25^ However, accurately predicting this benefit remains a significant challenge. Risk scores such as the APPLE score and CAAP-AF score, designed to predict AF recurrence after ablation, have demonstrated poor performance.^26,27^ Publications aimed at studying patient selection strategies for PVI or for additive substrate ablation are scarce. In the DECAAF II trial, pre-ablation MRI guided fibrosis ablation combined with PVI showed no significant difference in atrial arrhythmia recurrence compared to PVI alone in PeAF patients.^12^ This suggests that pre-ablation MRI is not effective at selecting patients to undergo additional ablation beyond PVI. It may be possible that the much more accessible strategy of rhythm monitoring following ECV could guide the selection of patients to undergo additional ablation beyond PVI, which is investigated in the ongoing PACIFIC trial (NCT05264831).^28^

According to a recent EHRA survey asking physicians which factors they consider during patient selection, more than 80% take into account left atrial size, age, severity of AF symptoms, coexistence of heart failure, and the presence of obesity; whereas only 42% consider previous response to ECV.^13^ According to the results of our study, the response to ECV is a strong predictor of the long-term efficacy of ablation of PeAF when keeping all other variables (including age and left atrial size) constant. Such a risk stratification approach could have a wide range of clinical applications to tailor treatment strategies to individual patients. If the decision is to ablate ECV-AF patients, it might be reasonable to consider more extensive ablation techniques beyond PVI. This also has implications for procedural planning, as these patients might be more suitable for an approach with systematic substrate mapping, and substrate modification (when appropriate) rather than a single-shot PVI-only procedure. Other potential clinical applications might include informing the timing of ablation, which ideally should be performed in ECV-AF patients as early as possible before further atrial remodelling develops. ECV response could also help in guiding post-procedural management. In this cohort, 25% of ECV-AF patients had an early recurrence in the first 4–5 months after ablation, while the early recurrence rate in ECV-SR patients was 0%. This suggests that the selection of patients for short-term post-procedural AAD therapy based on ECV response might be effective to reduce the number of early cardioversions and arrhythmia-related hospitalizations. Finally, this approach could support shared decision-making and effective communication with the patient about the expected benefits of the procedure.

Limitations of this study need to be mentioned. Due to its retrospective nature, ablation techniques and follow-up methods were not standardized during the study period, and unmeasured confounders may have impacted the results. To mitigate confounding and bias, stringent inclusion and exclusion criteria, PSM, and multi-variable regression methods were employed. This retrospective study does not identify the exact mechanisms underlying AF recurrences after ECV as substrate mapping or trigger induction and localization were not part of procedural protocols.

## Conclusion

The recurrence rates of atrial arrhythmias were higher in ECV-AF than in ECV-SR patients. Results from this propensity score-matched cohort suggest that the response to pre-procedural ECV may serve as a valuable marker for identifying persistent AF patients in whom a PVI-only strategy is sufficient.

## Lead author biography


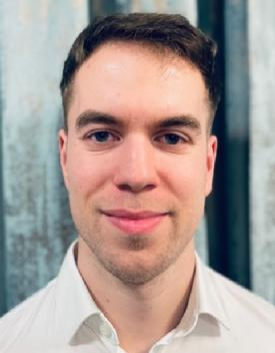


Dr Márton Boga received his medical degree at Semmelweis University in 2024 and completed the Clinical Science Scholars Programme at Harvard Medical School. Dr Boga is involved in clinical research as a PhD candidate under the supervision of Dr Nándor Szegedi at the Heart and Vascular Center, Budapest, Hungary. Their research focuses on cardiac electrophysiology, with a particular interest in catheter ablation techniques for atrial fibrillation. Outside of medicine, he is an avid ice hockey player and passionate windsurfer.

## Supplementary Material

oeaf084_Supplementary_Data

## Data Availability

Data underlying results presented in this paper will be made available upon request from the corresponding author.

## References

[1] Blum S, Meyre P, Aeschbacher S, Berger S, Auberson C, Briel M, Osswald S, Conen D. Incidence and predictors of atrial fibrillation progression: a systematic review and meta-analysis. Heart Rhythm 2019;16:502–510. 30366160 10.1016/j.hrthm.2018.10.022

[2] Steinberg BA, Hellkamp AS, Lokhnygina Y, Patel MR, Breithardt G, Hankey GJ, Becker RC, Singer DE, Halperin JL, Hacke W, Nessel CC, Berkowitz SD, Mahaffey KW, Fox KAA, Califf RM, Piccini JP. Higher risk of death and stroke in patients with persistent vs. Paroxysmal atrial fibrillation: results from the ROCKET-AF trial. Eur Heart J 2015;36:288–296. 25209598 10.1093/eurheartj/ehu359 PMC4313363

[3] Link MS, Giugliano RP, Ruff CT, Scirica BM, Huikuri H, Oto A, Crompton AE, Murphy SA, Lanz H, Mercuri MF, Antman EM, Braunwald E. Stroke and mortality risk in patients with Various patterns of atrial fibrillation. Circ Arrhythm Electrophysiol 2017;10:e004267. 28077507 10.1161/CIRCEP.116.004267

[4] Chew DS, Li Z, Steinberg BA, O'Brien EC, Pritchard J, Bunch TJ, Mark DB, Patel MR, Nabutovsky Y, Greiner MA, Piccini JP. Arrhythmic burden and the risk of cardiovascular outcomes in patients with paroxysmal atrial fibrillation and cardiac implanted electronic devices. Circ Arrhythm Electrophysiol 2022;15:e010304. 35089799 10.1161/CIRCEP.121.010304

[5] Van Gelder IC, Rienstra M, Bunting KV, Casado-Arroyo R, Caso V, Crijns HJGM, De Potter TJR, Dwight J, Guasti L, Hanke T, Jaarsma T, Lettino M, Løchen M-L, Lumbers RT, Maesen B, Mølgaard I, Rosano GMC, Sanders P, Schnabel RB, Suwalski P, Svennberg E, Tamargo J, Tica O, Traykov V, Tzeis S, Kotecha D, Dagres N, Rocca B, Ahsan S, Ameri P, Arbelo E, Bauer A, Borger MA, Buccheri S, Casadei B, Chioncel O, Dobrev D, Fauchier L, Gigante B, Glikson M, Hijazi Z, Hindricks G, Husser D, Ibanez B, James S, Kaab S, Kirchhof P, Køber L, Koskinas KC, Kumler T, Lip GYH, Mandrola J, Marx N, Mcevoy JW, Mihaylova B, Mindham R, Muraru D, Neubeck L, Nielsen JC, Oldgren J, Paciaroni M, Pasquet AA, Prescott E, Rega F, Rossello FJ, Rucinski M, Salzberg SP, Schulman S, Sommer P, Svendsen JH, ten Berg JM, Ten Cate H, Vaartjes I, Vrints CJ, Witkowski A, Zeppenfeld K, Simoni L, Kichou B, Sisakian HS, Scherr D, Cools F, Smajić E, Shalganov T, Manola S, Avraamides P, Taborsky M, Brandes A, El-Damaty AM, Kampus P, Raatikainen P, Garcia R, Etsadashvili K, Eckardt L, Kallergis E, Gellér L, Guðmundsson K, Lyne J, Marai I, Colivicchi F, Abdrakhmanov AS, Bytyci I, Kerimkulova A, Kupics K, Refaat M, Bheleel OA, Barysienė J, Leitz P, Sammut MA, Grosu A, Pavlovic N, Moustaghfir A, Yap S-C, Taleski J, Fink T, Kazmierczak J, Sanfins VM, Cozma D, Zavatta M, Kovačević DV, Hlivak P, Zupan I, Calvo D, Björkenheim A, Kühne M, Ouali S, Demircan S, Sychov OS, Ng A, Kuchkarov H. 2024 ESC guidelines for the management of atrial fibrillation developed in collaboration with the European association for cardio-thoracic surgery (EACTS): developed by the task force for the management of atrial fibrillation of the European Society of Cardiology (ESC), with the special contribution of the European heart rhythm association (EHRA) of the ESC. Endorsed by the European stroke organisation (ESO). Eur Heart J 2024;45:3314–3414. 39210723 10.1093/eurheartj/ehae176

[6] Tzeis S, Gerstenfeld EP, Kalman J, Saad EB, Sepehri Shamloo A, Andrade JG, Barbhaiya CR, Baykaner T, Boveda S, Calkins H. 2024 European heart rhythm association/heart rhythm society/Asia Pacific heart rhythm society/Latin American heart rhythm society expert consensus statement on catheter and surgical ablation of atrial fibrillation. EP Europace 2024;26:27–28. 10.1093/europace/euae043 PMC11000153 38587017

[7] Verma A, Jiang CY, Betts TR, Chen J, Deisenhofer I, Mantovan R, Macle L, Morillo CA, Haverkamp W, Weerasooriya R, Albenque J-P, Nardi S, Menardi E, Novak P, Sanders P. Approaches to catheter ablation for persistent atrial fibrillation. N Engl J Med 2015;372:1812–1822. 25946280 10.1056/NEJMoa1408288

[8] Huo Y, Gaspar T, Schönbauer R, Wójcik M, Fiedler L, Roithinger FX, Martinek M, Pürerfellner H, Kirstein B, Richter U, Ulbrich S, Mayer J, Krahnefeld O, Agdirlioglu T, Zedda A, Piorkowski J, Piorkowski C. Low-voltage myocardium-guided ablation trial of persistent atrial fibrillation. NEJM Evid 2022;1:EVIDoa2200141. 38319851 10.1056/EVIDoa2200141

[9] Valderrábano M, Peterson LE, Swarup V, Schurmann PA, Makkar A, Doshi RN, DeLurgio D, Athill CA, Ellenbogen KA, Natale A, Koneru J, Dave AS, Giorgberidze I, Afshar H, Guthrie ML, Bunge R, Morillo CA, Kleiman NS. Effect of catheter ablation with vein of Marshall ethanol infusion vs catheter ablation alone on persistent atrial fibrillation: the VENUS randomized clinical trial. JAMA 2020;324:1620–1628. 33107945 10.1001/jama.2020.16195 PMC7592031

[10] Kistler PM, Chieng D, Sugumar H, Ling L-H, Segan L, Azzopardi S, Al-Kaisey A, Parameswaran R, Anderson RD, Hawson J, Prabhu S, Voskoboinik A, Wong G, Morton JB, Pathik B, McLellan AJ, Lee G, Wong M, Finch S, Pathak RK, Raja DC, Sterns L, Ginks M, Reid CM, Sanders P, Kalman JM. Effect of catheter ablation using pulmonary vein isolation with vs without posterior left atrial wall isolation on atrial arrhythmia recurrence in patients with persistent atrial fibrillation: the CAPLA randomized clinical trial. JAMA 2023;329:127–135. 36625809 10.1001/jama.2022.23722 PMC9856612

[11] Biase LD, Burkhardt JD, Mohanty P, Mohanty S, Sanchez JE, Trivedi C, Güneş M, Gökoğlan Y, Gianni C, Horton RP, Themistoclakis S, Gallinghouse GJ, Bailey S, Zagrodzky JD, Hongo RH, Beheiry S, Santangeli P, Casella M, Dello Russo A, Al-Ahmad A, Hranitzky P, Lakkireddy D, Tondo C, Natale A. Left atrial appendage isolation in patients with longstanding persistent AF undergoing catheter ablation. J Am Coll Cardiol 2016;68:1929–1940. 27788847 10.1016/j.jacc.2016.07.770

[12] Marrouche NF, Wazni O, McGann C, Greene T, Dean JM, Dagher L, Kholmovski E, Mansour M, Marchlinski F, Wilber D, Hindricks G, Mahnkopf C, Wells D, Jais P, Sanders P, Brachmann J, Bax JJ, Morrison-de Boer L, Deneke T, Calkins H, Sohns C, Akoum N. Effect of MRI-guided fibrosis ablation vs conventional catheter ablation on atrial arrhythmia recurrence in patients with persistent atrial fibrillation: the DECAAF II randomized clinical trial. JAMA 2022;327:2296–2305. 35727277 10.1001/jama.2022.8831 PMC9214588

[13] Iliodromitis K, Lenarczyk R, Scherr D, Conte G, Farkowski MM, Marin F, Garcia-Seara J, Simovic S, Potpara T. Patient selection, peri-procedural management, and ablation techniques for catheter ablation of atrial fibrillation: an EHRA survey. EP Europace 2023;25:667–675. 10.1093/europace/euac236 PMC9935016 36512365

[14] Rivard L, Hocini M, Rostock T, Cauchemez B, Forclaz A, Jadidi AS, Linton N, Nault I, Miyazaki S, Liu X, Xhaet O, Shah A, Sacher F, Derval N, Jaïs P, Khairy P, Macle L, Nattel S. Improved outcome following restoration of sinus rhythm prior to catheter ablation of persistent atrial fibrillation: a comparative multicenter study. Heart Rhythm 2012;9:1025–1030. 22342863 10.1016/j.hrthm.2012.02.016

[15] Kamada H, Mori K, Ueda N, Wakamiya A, Nakajima K, Kamakura T, Wada M, Ishibashi K, Yamagata K, Inoue Y, Miyamoto K, Nagase S, Noda T, Izumi C, Noguchi T, Kusano K, Aiba T. Impact of Pre-ablation direct current cardioversion for persistent atrial fibrillation to predict recurrence of atrial fibrillation after catheter ablation. Int Heart J 2022;63:828–836. 36184544 10.1536/ihj.22-135

[16] Limite LR, Laborie G, Ramirez FD, Albenque JP, Combes S, Lagrange P, Khoueiry Z, Bortone A. Maintenance of sinus rhythm after electrical cardioversion to identify patients with persistent atrial fibrillation who respond favorably to pulmonary vein isolation: the pre-Pacific study. Front Cardiovasc Med 2024;11:1416975. 39465134 10.3389/fcvm.2024.1416975 PMC11502360

[17] Kang JH, Lee DI, Kim S, Kim MN, Park YM, Ban JE, Choi JI, Lim HE, Park SW, Kim YH. Prediction of long-term outcomes of catheter ablation of persistent atrial fibrillation by parameters of preablation DC cardioversion. J Cardiovasc Electrophysiol 2012;23:1165–1170. 22882453 10.1111/j.1540-8167.2012.02339.x

[18] Okawa K, Hara S, Morimoto T, Tsushima R, Sudo Y, Sogo M, Ozaki M, Takahashi M, Doi M, Morita H, Ito H. Effect of preprocedural pharmacologic cardioversion on pulmonary vein isolation in patients with persistent atrial fibrillation. Heart Rhythm 2021;18:1473–1479. 33932587 10.1016/j.hrthm.2021.04.027

[19] Eberly LA, Lin A, Park J, Khoshnab M, Garg L, Chee J, Kallan MJ, Walsh K, Supple GE, Schaller RD, Santangeli P, Riley MP, Nazarian S, Arkles J, Hyman M, Lin D, Guandalini G, Kumareswaran R, Deo R, Zado ES, Epstein A, Frankel DS, Callans DJ, Marchlinski FE, Dixit S. Presence of sinus rhythm at time of ablation in patients with persistent atrial fibrillation undergoing pulmonary vein isolation is associated with improved long-term arrhythmia outcomes. J Interv Card Electrophysiol 2023;66:1455–1464. 36525168 10.1007/s10840-022-01441-4

[20] Link MS, Haïssaguerre M, Natale A. Ablation of atrial fibrillation: patient selection, periprocedural anticoagulation, techniques, and preventive measures after ablation. Circulation 2016;134:339–352. 27462054 10.1161/CIRCULATIONAHA.116.021727

[21] Szegedi N, Vecsey-Nagy M, Simon J, Szilveszter B, Herczeg S, Kolossváry M, Idelbi H, Osztheimer I, Klaudia Nagy V, Tahin T, Széplaki G, Delgado V, Bax JJ, Maurovich-Horvat P, Merkely B, Gellér L. Orientation of the right superior pulmonary vein affects outcome after pulmonary vein isolation. Eur Heart J Cardiovasc Imaging 2022;23:515–523. 33693618 10.1093/ehjci/jeab041

[22] Szegedi N, Simon J, Szilveszter B, Salló Z, Herczeg S, Száraz L, Kolossváry M, Orbán G, Széplaki G, Nagy KV, Mahdiui ME, Smit JM, Delgado V, Bax JJ, Maurovich-Horvat P, Merkely B, Gellér L. Abutting left atrial appendage and left superior pulmonary vein predicts recurrence of atrial fibrillation after point-by-point pulmonary vein isolation. Front Cardiovasc Med 2022;9:708298. 35242821 10.3389/fcvm.2022.708298 PMC8885731

[23] Simon J, El Mahdiui M, Smit JM, Száraz L, van Rosendael AR, Herczeg S, Zsarnóczay E, Nagy AI, Kolossváry M, Szilveszter B, Szegedi N, Nagy KV, Tahin T, Gellér L, van der Geest RJ, Bax JJ, Maurovich-Horvat P, Merkely B. Left atrial appendage size is a marker of atrial fibrillation recurrence after radiofrequency catheter ablation in patients with persistent atrial fibrillation. Clin Cardiol 2022;45:273–281. 34799870 10.1002/clc.23748 PMC8922535

[24] Tóth P, Arnóth B, Komlósi F, Szegedi N, Salló Z, Perge P, Osztheimer I, Merkely B, Gellér L, Nagy KV. Effect of early catheter ablation of atrial fibrillation in patients with heart failure. J Cardiovasc Electrophysiol 2024;35:1471–1479. 38803006 10.1111/jce.16300

[25] El Mahdiui M, Simon J, Smit JM, Kuneman JH, van Rosendael AR, Steyerberg EW, van der Geest RJ, Száraz L, Herczeg S, Szegedi N, Gellér L, Delgado V, Merkely B, Bax JJ, Maurovich-Horvat P. Posterior left atrial adipose tissue attenuation assessed by computed tomography and recurrence of atrial fibrillation after catheter ablation. Circ Arrhythm Electrophysiol 2021;14:e009135. 33720759 10.1161/CIRCEP.120.009135

[26] Kornej J, Hindricks G, Shoemaker MB, Husser D, Arya A, Sommer P, Rolf S, Saavedra P, Kanagasundram A, Patrick Whalen S, Montgomery J, Ellis CR, Darbar D, Bollmann A. The APPLE score: a novel and simple score for the prediction of rhythm outcomes after catheter ablation of atrial fibrillation. Clin Res Cardiol 2015;104:871–876. 25876528 10.1007/s00392-015-0856-x PMC4726453

[27] Winkle RA, Jarman JW, Mead RH, Engel G, Kong MH, Fleming W, Patrawala RA. Predicting atrial fibrillation ablation outcome: the CAAP-AF score. Heart Rhythm 2016;13:2119–2125. 27435586 10.1016/j.hrthm.2016.07.018

[28] Bortone AA, Marijon E, Limite LR, Lagrange P, Brigadeau F, Martins R, Durand C, Albenque J-P, group ftPs. Pulmonary vein isolation alone or in combination with substrate modulation after electrical cardioversion failure in patients with persistent atrial fibrillation: the PACIFIC trial: study design. J Cardiovasc Electrophysiol 2023;34:270–278. 36434797 10.1111/jce.15761

