# Supplementary material for: Impact of response to electrical cardioversion before catheter ablation for persistent atrial fibrillation: a propensity score-matched analysis
Source: Eur Heart J Open. 2025 Jun 28;5(4):oeaf084. doi: 10.1093/ehjopen/oeaf084 (PMC12264425; doi:10.1093/ehjopen/oeaf084)
Supplement: oeaf084_Supplementary_Data [file oeaf084_supplementary_data.docx]

**SUPPLEMENTARY MATERIAL**

Supplementary table 1. Baseline characteristics of unmatched groups. AF = atrial fibrillation, BMI = body-mass index, CAD = coronary artery disease, CFAE = complex fractionated atrial electrogram, CHF = chronic heart failure, CTI = cavotricuspid isthmus, ECV = electrical cardioversion, LAD = left atrial diameter, LVEF = left ventricular ejection fraction, PAD = peripheral arterial disease, PVI = pulmonary vein isolation, TIA = transient ischemic attack, VHD = valvular heart disease. Bold P-values indicate significance at alpha<0.05.

|  | **ECV-SR**  **N = 94 (43%)** | **ECV-AF**  **N = 125 (57%)** | **P-value** |
| --- | --- | --- | --- |
| Age, years | 63.5 (55 – 69) | 63 (54 – 70) | 0.853 |
| Female sex, N (%) | 23 (24.5%) | 45 (36.0%) | 0.068 |
| BMI, kg/m^2^ | 29.3 (27.3–32.1) | 29.0 (26.1–33.0) | 0.778 |
| AF pattern  Persistent, N (%)  Long-standing persistent, N (%) | 89 (95)  5 (5) | 105 (84)  20 (16) | **0.014** |
| Hypertension, N (%) | 56 (59.6%) | 96 (76.8%) | **0.006** |
| CHF, N (%) | 12 (12.8%) | 15 (12.0%) | 0.864 |
| CAD, N (%) | 9 (9.6%) | 21 (16.8%) | 0.124 |
| VHD, N (%) | 6 (6.4%) | 11 (8.8%) | 0.508 |
| Stroke/TIA, N (%) | 7 (7.4%) | 4 (3.2%) | 0.154 |
| Diabetes, N (%) | 14 (14.9%) | 28 (22.4%) | 0.163 |
| PAD, N (%) | 2 (2.1%) | 3 (2.4%) | 0.894 |
| Hypertyreosis, N (%) | 0 (0.0%) | 8 (6.4%) | **0.012** |
| Left atrial diameter, mm | 49 (45.5 – 52) | 48 (42 – 51.5) | 0.361 |
| Right atrial diameter, mm | 45.4 (41.5–49) | 45.5 (38.25–51) | 0.946 |
| E wave velocity, cm/s | 80 (63–98) | 93 (78–100) | **0.002** |
| LAA flow velocity, cm/s | 39 (26–52) | 30 (25–40) | **0.018** |
| LVEF, % | 55 (55–60) | 55 (54–56) | 0.234 |
| LAVI, ml/m^2^ | 46.1 (34.7–58.9) | 46.2 (36.3–58.3) | 0.981 |
| CHA_2_DS_2_-VA score, N (%)  0  1  2  3  4  5  6 | 22 (23)  26 (28)  24 (26)  15 (16)  7 (7)  0 (0)  0 (0) | 18 (14)  36 (29)  33 (26)  23 (18)  9 (7)  5 (4)  1 (1) | **0.030** |
| ECV-to-PVI time, days | 99.5 (71 – 130) | 98 (68 – 132) | 0.749 |
| Date of ablation procedure, year | 2018 (2016–2021) | 2016 (2015–2019) | **<0.001** |
| PT, min | 80 (70 – 110) | 90 (75 – 110) | 0.291 |
| LA dwell time, min | 56.5 (43 – 75.5) | 58.5 (44 – 74) | 0.861 |
| Additional ablation, N (%)  Posterior wall box lesion  Mitral isthmus line  CTI line  CFAE ablation | 6 (6)  3 (3)  2 (2)  3 (3)  0 (0) | 44 (35)  28 (22)  16 (13)  6 (5)  2 (2) | **<0.001** |
| Follow-up duration, months | 42 (22–71) | 40 (15–76) | 0.584 |

Supplementary table 2. Univariable Cox-regression estimates for matched population. AF = atrial fibrillation, AT = atrial tachycardia, CAD = coronary artery disease, CHF = chronic heart failure, CI = confidence interval, ECV = electrical cardioversion, HR = hazard ratio, LAD = left atrial diameter, LVEF = left ventricular ejection fraction, PAD = peripheral arterial disease, TIA = transient inchemic attack, VHD = valvular heart disease.

|  | **Endpoint of AF/AT recurrence** | | **Endpoint of AF recurrence in persistent form** | |
| --- | --- | --- | --- | --- |
|  | **HR**  **(95% CI)** | **P-value** | **HR**  **(95% CI)** | **P-value** |
| Age, years | 0.99  (0.98–1.02) | 0.882 | 0.97  (0.94–1.00) | 0.092 |
| Female sex | 1.33  (0.87 - 2.04) | 0.188 | 1.14  (0.61 - 2.13) | 0.672 |
| Hypertension | 0.86  (0.56 - 1.33) | 0.497 | 0.80  (0.41 - 1.57) | 0.517 |
| CHF | 1.40  (0.79 - 2.49) | 0.25 | 1.97  (0.86 - 4.53) | 0.109 |
| CAD | 0.81  (0.42 - 1.58) | 0.542 | 0.73  (0.28 - 1.88) | 0.513 |
| VHD | 1.76  (0.81 - 3.82) | 0.156 | 2.27  (0.68 - 7.55) | 0.183 |
| Stroke/TIA | 1.16  (0.53 - 2.52) | 0.716 | 0.94  (0.22 - 3.97) | 0.936 |
| Diabetes | 2.15  (1.30 - 3.57) | 0.003 | 0.97  (0.45 - 2.09) | 0.931 |
| PAD | 0.29  (0.04 - 2.13) | 0.225 | 0 | 1 |
| Long-standing persistent AF | 2.06  (0.94 - 4.54) | 0.072 | 1.77  (0.54 - 5.85) | 0.347 |
| LVEF, % | 0.97  (0.95 - 0.99) | 0.027 | 0.95  (0.92 - 0.99) | 0.005 |
| LAD, mm | 1.01  (0.98 - 1.03) | 0.701 | 1.02  (0.98 - 1.07) | 0.363 |
| Additional ablation | 1.45  (0.89 - 2.36) | 0.133 | 0.82  (0.35 - 1.90) | 0.645 |
| CHA_2_DS_2_-VA | 1.09  (0.92 - 1.28) | 0.328 | 0.93  (0.71 - 1.21) | 0.567 |
| ECV-AF | 2.42  (1.61 - 3.65) | <0.001 | 3.72  (1.94 - 7.14) | <0.001 |


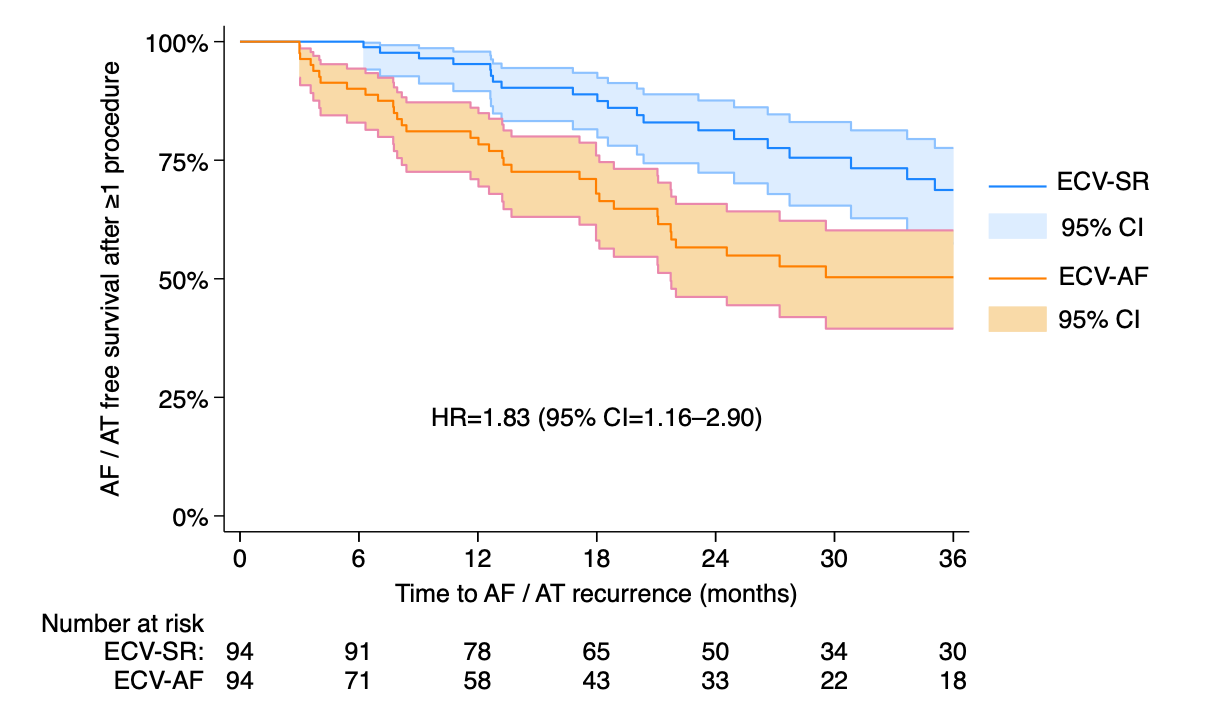


Supplementary Figure 1. Kaplan-Meier curves of AF / AT free survival after one or more procedures. AF = atrial fibrillation, AT = atrial tachycardia, CI = confidence interval, HR = hazard ratio.
